# Intrinsic Neuronal Properties Switch the Mode of Information Transmission in Networks

**DOI:** 10.1371/journal.pcbi.1003962

**Published:** 2014-12-04

**Authors:** Julijana Gjorgjieva, Rebecca A. Mease, William J. Moody, Adrienne L. Fairhall

**Affiliations:** 1 Center for Brain Science, Harvard University, Cambridge, Massachusetts, United States of America; 2 Institute of Neuroscience, Technische Universität München, Munich, Germany; 3 Department of Biology, University of Washington, Seattle, Washington, United States of America; 4 Department of Physiology and Biophysics and the WRF UW Institute for Neuroengineering, University of Washington, Seattle, Washington, United States of America; Philipps-University Marburg, Germany

## Abstract

Diverse ion channels and their dynamics endow single neurons with complex biophysical properties. These properties determine the heterogeneity of cell types that make up the brain, as constituents of neural circuits tuned to perform highly specific computations. How do biophysical properties of single neurons impact network function? We study a set of biophysical properties that emerge in cortical neurons during the first week of development, eventually allowing these neurons to adaptively scale the gain of their response to the amplitude of the fluctuations they encounter. During the same time period, these same neurons participate in large-scale waves of spontaneously generated electrical activity. We investigate the potential role of experimentally observed changes in intrinsic neuronal properties in determining the ability of cortical networks to propagate waves of activity. We show that such changes can strongly affect the ability of multi-layered feedforward networks to represent and transmit information on multiple timescales. With properties modeled on those observed at early stages of development, neurons are relatively insensitive to rapid fluctuations and tend to fire synchronously in response to wave-like events of large amplitude. Following developmental changes in voltage-dependent conductances, these same neurons become efficient encoders of fast input fluctuations over few layers, but lose the ability to transmit slower, population-wide input variations across many layers. Depending on the neurons' intrinsic properties, noise plays different roles in modulating neuronal input-output curves, which can dramatically impact network transmission. The developmental change in intrinsic properties supports a transformation of a networks function from the propagation of network-wide information to one in which computations are scaled to local activity. This work underscores the significance of simple changes in conductance parameters in governing how neurons represent and propagate information, and suggests a role for background synaptic noise in switching the mode of information transmission.

## Author Summary

Differences in ion channel composition endow different neuronal types with distinct computational properties. Understanding how these biophysical differences affect network-level computation is an important frontier. We focus on a set of biophysical properties, experimentally observed in developing cortical neurons, that allow these neurons to efficiently encode their inputs despite time-varying changes in the statistical context. Large-scale propagating waves are autonomously generated by the developing brain even before the onset of sensory experience. Using multi-layered feedforward networks, we examine how changes in intrinsic properties can lead to changes in the network's ability to represent and transmit information on multiple timescales. We demonstrate that measured changes in the computational properties of immature single neurons enable the propagation of slow-varying wave-like inputs. In contrast, neurons with more mature properties are more sensitive to fast fluctuations, which modulate the slow-varying information. While slow events are transmitted with high fidelity in initial network layers, noise degrades transmission in downstream network layers. Our results show how short-term adaptation and modulation of the neurons' input-output firing curves by background synaptic noise determine the ability of neural networks to transmit information on multiple timescales.

## Introduction

*Gain scaling* refers to the ability of neurons to scale the gain of their responses when stimulated with currents of different amplitudes. A common property of neural systems, gain scaling adjusts the system's response to the size of the input relative to the input's standard deviation [1]. This form of adaptation maximizes information transmission for different input distributions [1]–[3]. Though this property is typically observed with respect to the coding of external stimuli by neural circuits [1], [3]–[7], Mease *et al.* [8] have recently shown that single neurons during early development of mouse cortex automatically adjust the dynamic range of coding to the scale of input stimuli through a modulation of the slope of their effective input-output relationship. In contrast to previous work, perfect gain scaling in the input-output relation occurs for certain values of ionic conductances and does not require any explicit adaptive processes that adjust the gain through spike-driven negative feedback, such as slow sodium inactivation [4], [9], [10] and slow afterhyperpolarization (AHP) currents [10], [11]. However, these experiments found that gain scaling is not a static property during development. At birth, or P0 (postnatal day 0), cortical neurons show limited gain scaling; in contrast, at P8, neurons showed pronounced gain-scaling abilities [8]. Here, we examined how the emergence of the gain-scaling property in single cortical neurons during the first week of development might affect signal transmission over multiple timescales across the cortical network.

Along with the emergence of gain scaling during the first week of neural development, single neurons in the developing cortex participate in large-scale spontaneously generated activity which travels across different regions in the form of waves [12]–[14]. *Pacemaker* neurons located in the ventrolateral (piriform) cortex initiate spontaneous waves that continue to propagate dorsally across the neocortex [13]. Experimentally, much attention has been focused on synaptic interactions in initiating and propagating activity, with a particular emphasis on the role of GABAergic circuits, which are depolarizing in early development [15], [16]. While multiple network properties play an important role in the generation of spontaneous waves, here we ask how the intrinsic computational properties of cortical neurons, in particular gain scaling, can affect the generation and propagation of spontaneous activity. Changes in intrinsic properties may play a role in wave propagation during development, and the eventual disappearance of this activity as sensory circuits become mature.

A simple model for propagating activity, like that observed during spontaneous waves, is a feedforward network in which activity is carried from one population, or layer, of neurons to the next without affecting previous layers [17]. We compare the behavior of networks composed of conductance-based neurons with either immature (nongain-scaling) or mature (gain-scaling) computational properties [8]. These networks exhibit different information processing properties with respect to both fast and slow timescales of the input. We determine how rapid input fluctuations are encoded in the precise spike timing of the output by the use of linear-nonlinear models [18], [19], and use noise-modulated frequency-current relationships to predict the transmission of slow variations in the input [20], [21].

We find that networks built from neuron types with different gain-scaling ability propagate information in strikingly different ways. Networks of gain-scaling (GS) neurons convey a large amount of fast-varying information from neuron to neuron, and transmit slow-varying information at the population level, but only across a few layers in the network; over multiple layers the slow-varying information disappears. In contrast, nongain-scaling (NGS) neurons are worse at processing fast-varying information at the single neuron level; however, subsequent network layers transmit slow-varying signals faithfully, reproducing wave-like behavior. We qualitatively explain these results in terms of the differences in the noise-modulated frequency-current curves of the neuron types through a mean field approach: this approach allows us to characterize how the mean firing rate of a neuronal population in a given layer depends on the firing rate of the neuronal population in the previous layer through the mean synaptic currents exchanged between the two layers. Our results suggest that the experimentally observed changes in intrinsic properties may contribute to the transition from spontaneous wave propagation in developing cortex to sensitivity to local input fluctuations in more mature networks, priming cortical networks to become capable of processing functionally relevant stimuli.

## Results

Single cortical neurons acquire the ability to scale the gain of their responses in the first week of development, as shown in cortical slice experiments [8]. Here, we described gain scaling by characterizing a single neuron's response to white noise using *linear/nonlinear* (LN) models (see below). Before becoming efficient encoders of fast stimulus fluctuations, the neurons participate in network-wide activity events that propagate along stereotypical directions, known as spontaneous cortical waves [13], [22]. Although many parameters regulate these waves in the developing cortex, we sought to understand the effect of gain scaling in single neurons on the ability of cortical networks to propagate information about inputs over long timescales, as occur during waves, and over short timescales, as occur when waves disappear and single neurons become efficient gain scalers. More broadly, we use waves in developing cortex as an example of a broader issue: how do changes in intrinsic properties of biophysically realistic model neurons affect how a network of such neurons processes and transmits information?

We have shown that in cortical neurons in brain slices, developmental increases in the maximal sodium (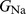) to potassium (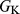) conductance ratio can explain the parallel transition from nongain-scaling to gain scaling behavior [8]. Furthermore, the gain scaling ability can be controlled by pharmacological manipulation of the maximal 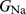 to 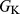 ratio [8]. The gain scaling property can also be captured by changing this ratio in single conductance-based model neurons [8]. Therefore, we first examined networks consisting of two types of neurons: where the ratio of 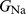 to 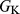 was set to either 0.6 (representing immature, nongain-scaling neurons) or 1.5 (representing mature, gain-scaling neurons).

### Two computational regimes at different temporal resolution

We first characterized neuronal responses of conductance-based model neurons using methods previously applied to experimentally recorded neurons driven with white noise. The neuron's *gain scaling* ability is defined by a rescaling of the input/output function of a linear/nonlinear (LN) model by the stimulus standard deviation [8]. Using a white noise input current, we extracted LN models describing the response properties of the two neuron types to *rapid fluctuations*, while fixing the mean (DC) of the input current. The LN model [18], [19], [23] predicts the instantaneous time-varying firing rate of a single neuron by first identifying a relevant feature of the input, and after linearly filtering the input stimulus with this feature, a nonlinear input-output curve that relates the magnitude of that feature in the input (the filtered stimulus) to the probability of firing. We computed the spike-triggered average (STA) as the relevant feature of the input [18], [24], and then constructed the nonlinear response function as the probability of firing given the stimulus linearly filtered by the STA.

**Figure 1:**
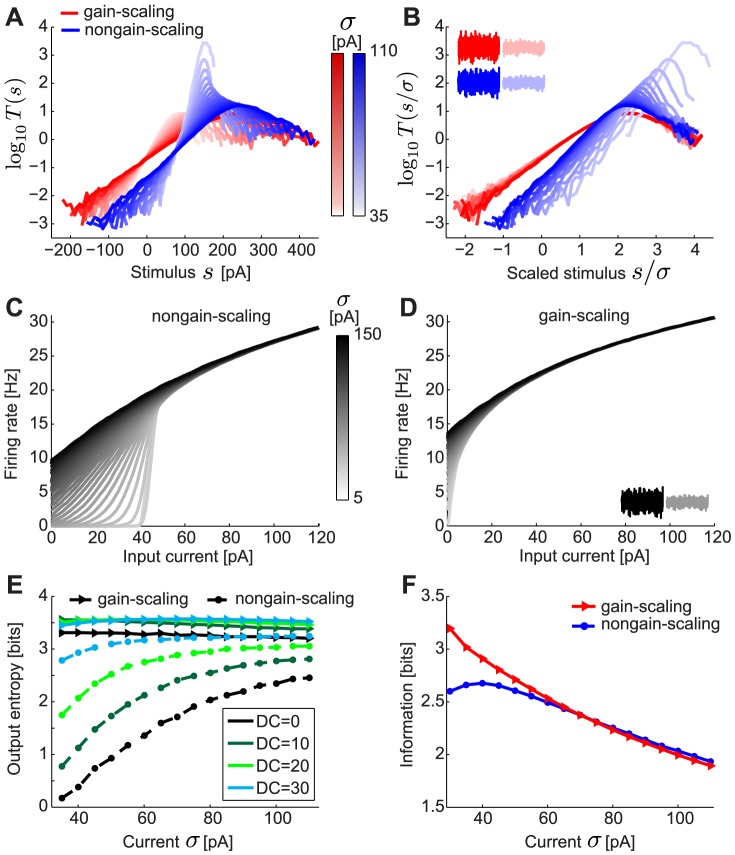
LN models and 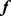
–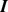 curves for gain-scaling (GS) and nongain-scaling (NGS) neurons. A. The nonlinearities in the LN model framework for a GS (red) (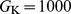 pS/µm^2^ and 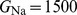 pS/µm^2^) and a NGS (blue) (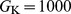 pS/µm^2^ and 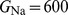 pS/µm^2^) neuron simulated as conductance-based model neurons (Eq. 2). The nonlinearities were computed using Bayes' rule: 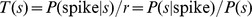, where 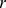 is the neuron's mean firing rate and 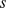 is the linearly filtered stimulus (see also Eq. 7 in Methods). B. The same nonlinearities as A, in stimulus units scaled by 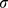 (magnitude of stimulus fluctuations). The nonlinearities overlap for GS neurons over a wide range of 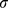. C–D. The 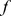
–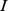 curves for a NGS (C) and a GS neuron (D) for different values of 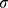. E. The output entropy as a function of the mean (DC) and 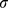 (amplitude of fast fluctuations). F. Information about the output firing rate of the neurons as a function of 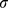.

Repeating this procedure for noise stimuli with a range of standard deviations (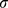) produces a family of curves for both neuron types (Figure 1A). While the linear feature is relatively constant as a function of the magnitude of the rapid fluctuations, 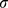, the nonlinear input-output curves change, similar to experimental observations in single neurons in cortical slices [8]. When the input is normalized by 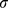, the *mature* neurons have a common input-output curve with respect to the normalized stimulus (Figure 1B, red) [8] over a wide range of input DC. In contrast, the input-output curves of *immature* neurons have a different slope when compared in units of the normalized stimulus (Figure 1B, blue). Gain scaling has previously been shown to support a high rate of information transmission about stimulus fluctuations in the face of changing stimulus amplitude [1]. Indeed, these GS neurons have higher output entropy, and therefore transmit more information, than NGS neurons (Figure 1E). The output entropy is approximately constant regardless of 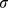 for a range of mean (DC) inputs – this is a hallmark of their gain-scaling ability. The changing shape of the input-output curve for the NGS neurons results in an increasing output entropy as a function of 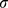 (Figure 1E). With the addition of DC, the output entropy of the NGS neurons' firing eventually approaches that of the GS neurons; this is accompanied with a simultaneous decrease in the distance between rest and threshold membrane potential of the NGS neurons as shown previously [8]. Thus, GS neurons are better at encoding fast fluctuations, a property which might enable efficient local computation independent of the background signal amplitude in more mature circuits after waves disappear.

The response of a neuron to slow input variations may be described in terms of its firing rate as a function of the mean input 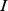 through a frequency-current (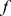
–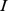) curve. This description averages over the details of the rapid fluctuations. The shape of this 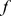
–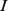 curve can be modulated by the standard deviation (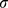) of the background noise [20], [21]. Here, the "background noise'' is a rapidly-varying input that is not considered to convey specific stimulus information but rather, provides a statistical context that modulates the signaled information assumed to be contained in the slow-varying mean input. Thus, a neuron's slow-varying responses can be characterized in terms of a family of 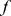
–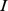 curves parameterized by 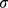.

Comparing the 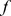
–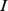 curves for the two neuron types using the same conductance-based models reveals substantial differences in their firing thresholds and also in their modulability by 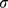 (Figure 1C,D). NGS neurons have a relatively high threshold at low 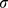, and the 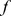
–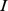 curves are significantly modulated by the addition of noise, i.e. with increasing 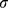 (Figure 1C). In contrast, the 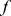
–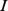 curves of GS neurons have lower thresholds, and show minimal modulation with the level of noise (Figure 1D). This behavior is reflected in the information that each neuron type transmits about firing rate for a range of 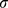 (Figure 1F). This information quantification determines how well a distribution of input DC can be distinguished at the level of the neuron's output firing rate while averaging out the fast fluctuations. The information would be low for neurons whose output firing rates are indistinguishable for a range of DC inputs, and high for neurons whose output firing rates unambiguously differ for different DC inputs. The two neuron types convey similar information for large 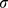 where the 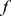
–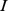 curves are almost invariant to noise magnitude. For GS neurons, most information is conveyed about the input rate at low 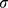 where the 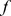
–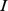 curve encodes the largest range of firing rates (0 to 30 Hz). The information encoded by NGS neurons is non-monotonic: at low 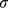 these neurons transmit less information because of their high thresholds, compressing the range of inputs being encoded. Information transmission is maximized at 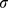 for which the 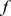
–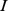 curve approaches linearity, simultaneously maximizing the range of inputs and outputs encoded by the neuron. For both neuron types, the general trend of decreasing information as 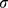 increases is the result of compressing the range of outputs (10 to 30 Hz).

These two descriptions characterize the different processing abilities of the two neuron types. GS neurons with their 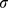-invariant input-output relations of the LN model are better suited to efficiently encode fast current fluctuations because information transmission is independent of 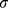. However, NGS neurons with their 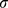-modulatable 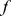
–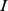 curves are better at representing a range of mean inputs, as illustrated by their ability to preserve the range of input currents in the range of output firing rates.

### The ratio of 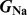 and 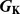 is sufficient for modulating a neuron's intrinsic computation

**Figure 2:**
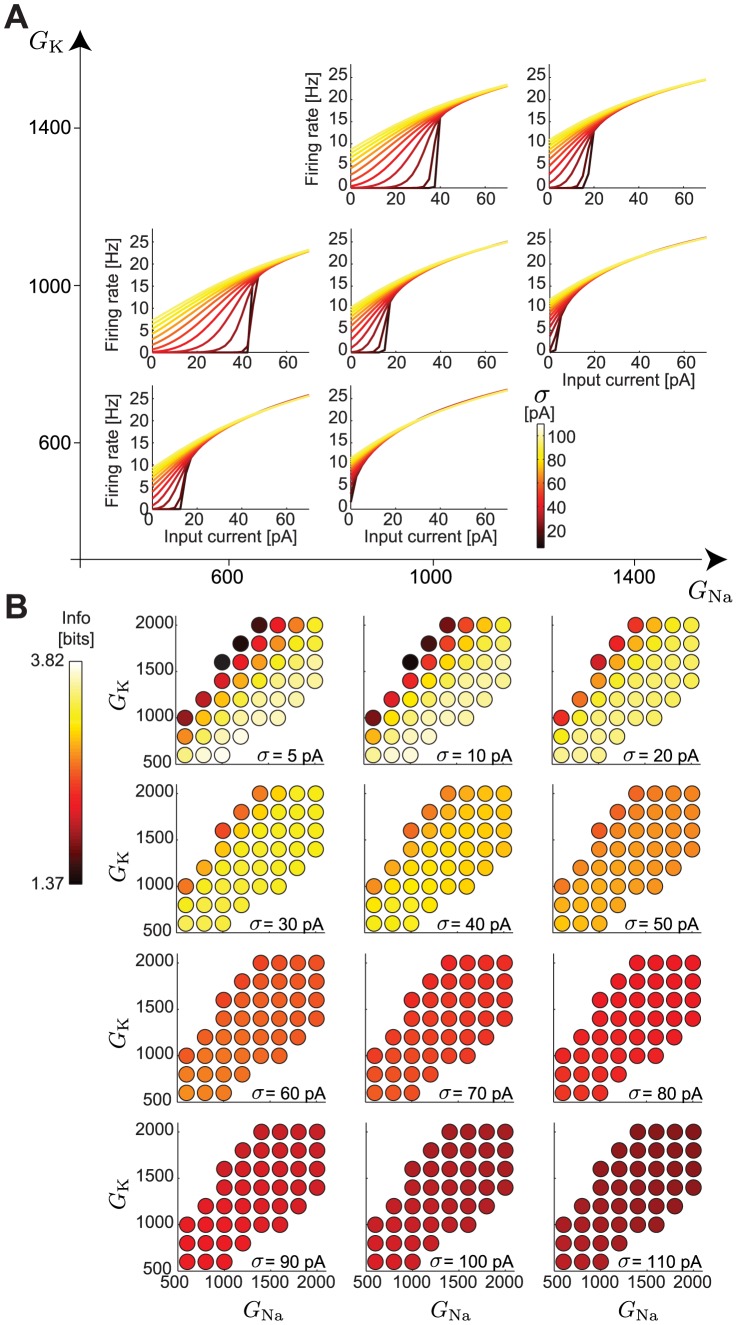
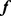
–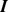 curves and information as a function of individual maximal Na and K conductances. A. The 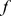
–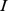 curves for different maximal Na and K conductances, 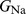 and 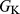, in pS/µm^2^ (compare to Figure 1C,D). B. The information for the different models as a function of 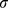 (compare to Figure 1F).

To characterize the spectrum of intrinsic properties that might arise as a result of different maximal conductances, 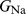 and 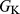, we determined the 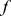
–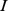 curves for a range of maximal conductances in the conductance-based model neurons (Figure 2). Mease et al. [8] previously classified neurons as spontaneously active, excitable or silent, and based on the neurons' LN models determined gain-scaling ability as a function of the individual 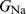 and 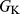 for excitable neurons. Models with low 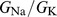 had nonlinear input-output relations that did not scale completely with 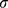, while models with high 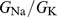 had almost identical nonlinear input-output relations for all 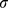 [8]. Therefore, gain scaling ability increased with increasing ratio, independent of each individual conductance.

We examined the modulability of 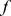
–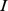 curves by 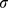 in excitable model neurons while independently varying 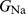 and 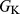 (Figure 2). Like gain scaling, the modulability by 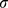 also depended only on the ratio 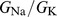, rather than either conductance alone, with larger modulability observed for smaller ratios. To further explore the implications of such modulability by 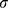, we computed the mutual information that each model neuron transmits about mean inputs for a range of 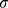 (Figure 2). Neurons with 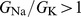 behaved like GS neurons in Figure 1F, while neurons with 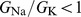 behaved like NGS neurons.

These results suggest that the ability of single neurons to represent a distribution of mean input currents by their distribution of output firing rates can be captured only by changing the ratio of 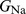 and 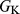. Therefore, we focused on studying two neuron types with 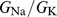 in the two extremes of the conductance range of excitable neurons: GS neurons with 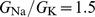 and NGS neurons with 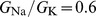.

### Population responses of the two neuron types

**Figure 3:**
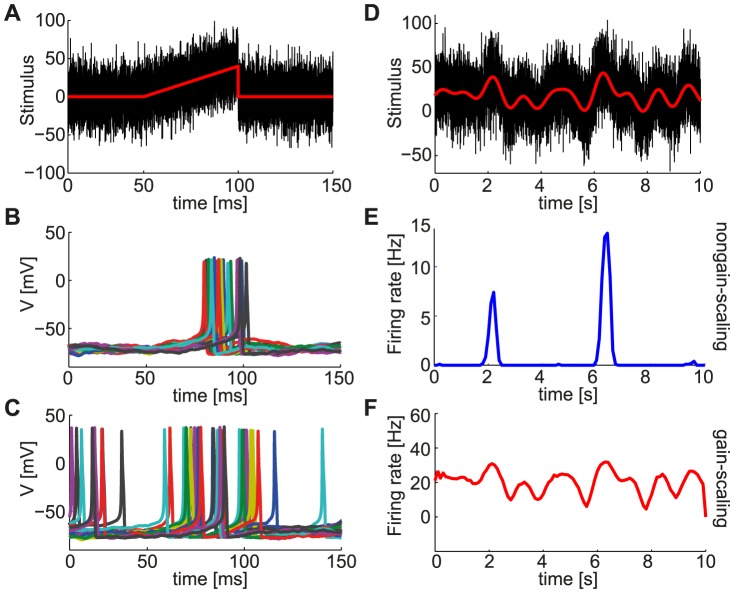
Stimulus encoding varies with the intrinsic properties of neurons. **A**. Noise fluctuations (black) superimposed on a short ramping input stimulus (red) with rise time of 50 ms were presented to two separate populations of 100 independent conductance-based model neurons with different gain-scaling properties. **B,C**. Voltage responses of (B) 100 NGS (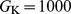 pS/µm^2^ and 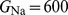 pS/µm^2^) and (C) 100 GS neurons (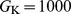 pS/µm^2^ and 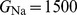 pS/µm^2^) to the ramp input in A. The different colors indicate voltage responses of different neurons. **D**. Noise fluctuations with a correlation time constant of 1 ms (black) superimposed on a Gaussian input stimulus low-pass filtered at 500 ms (red) for a duration of 10 seconds were also presented to the two neuron populations. **E,F**. Population response (PSTH) of NGS (E) and GS (F) neurons to the input in D.

Upon characterizing single neuron responses of the two neuron types to fast-varying information via the LN models and to slow-varying information via the 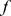
–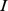 curves, we compared their population responses to stimuli with fast and slow timescales. A population of uncoupled neurons of each type was stimulated with a common slow ramp of input current, and superimposed fast-varying noise inputs, generated independently for each neuron (Figure 3A). The population of NGS neurons fired synchronously with respect to the ramp input and only during the peak of the ramp (Figure 3B), while the GS neurons were more sensitive to the background noise and fired asynchronously during the ramp (Figure 3C) with a firing rate that was continuously modulated by the ramp input. This suggests that the sensitivity to noise fluctuations of the GS neurons at the single neuron level allows them to better encode slower variations in the common signal at the population level [25]–[27], in contrast to the NGS population which only responds to events of large amplitude independent of the background noise.

During cortical development, wave-like activity on longer timescales occurs in the midst of fast-varying random synaptic fluctuations [13], [14], [28], [29]. Therefore, we compared the population responses of GS and NGS neurons to a slow-varying input (500 ms correlation time constant) common to all neurons with fast-varying background noise input (1 ms correlation time constant) independent for all neurons (Figure 3D). The distinction between the two neuron types is evident in the mean population responses (peristimulus time histogram, i.e. PSTH). The NGS population only captured the stimulus peaks (Figure 3E) while the GS population faithfully captured the temporal fluctuations of the common signal, aided by each neuron's temporal jitter caused by the independent noise fluctuations (Figure 3F). Although not an exact model of cortical wave development, this comparison supports the hypothesis that the intrinsic properties of single neurons can lead to different information transmission capabilities of cortical networks at different developmental time points, and the transition from wave propagation to wave cessation.

### Transmission of slow-varying information through the network

**Figure 4:**
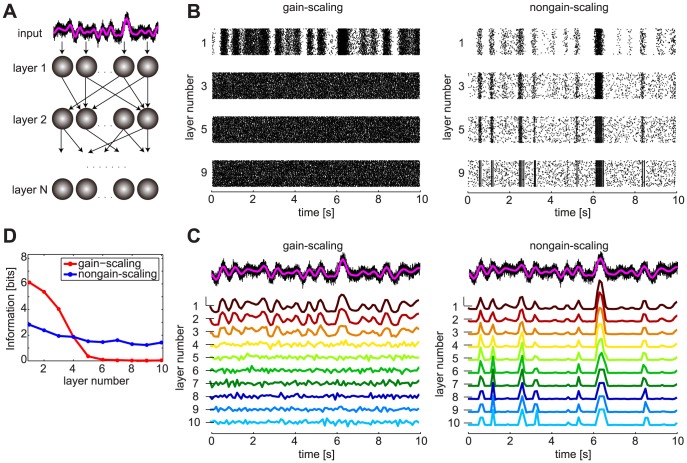
Information transmission through GS and NGS networks. **A**. Feedforward network with a slowly modulated time-varying input (magenta) presented to all neurons in the first layer, each neuron receiving in addition an independent noisy signal (black). **B**. Spike rasters for GS neurons (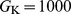 pS/µm^2^ and 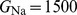 pS/µm^2^) show the rapid signal degradation in deeper layers, while NGS neurons (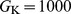 pS/µm^2^ and 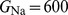 pS/µm^2^) exhibit reliable signal transmission of large-amplitude events. The spiking responses synchronize in deeper layers. **C**. PSTHs from each layer in the two networks showing the propagation of a slow-varying input in the presence of background fast fluctuations. PSTHs were normalized to mean 0 and variance 1 to illustrate fluctuations (in spite of different firing rates) so that the dashed lines next to each PSTH denote 0 and the scalebar 2 normalized units. **D**. Information about the slow stimulus fluctuations conveyed by the population mean responses shown in C.

The observed difference between the population responses of the GS and NGS neurons to the slow-varying stimulus in the presence of fast background fluctuations (Figure 3D–F) suggested that the two neuron types differ in their ability to transmit information at slow timescales. Therefore, we next examined how the identified single neuron properties affect information transmission across multiple layers in feedforward networks. Networks consisted of 10 layers of 2000 identical neurons of the two different types (Figure 4A). The neurons in the first layer receive a common temporally fluctuating stimulus with a long correlation time constant (1 s, see Methods); neurons in deeper layers receive synaptic input from neurons in the previous layer via conductance-based synapses. Each neuron in the network also receives a rapidly varying independent noise input (with a correlation time constant of 1 ms) to simulate fast-varying synaptic fluctuations. The noise input here is a rapidly-varying input that sets the statistical context for the slow-varying information; it does not transmit specific stimulus information itself. The GS and NGS networks have strikingly different spiking dynamics (Figure 4B). The GS network responds with higher mean firing rates in each layer, as would be expected from the 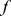
–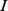 curves characterizing intrinsic neuronal properties (Figure 1C,D). While the GS neurons have a baseline firing rate even at zero input current, the NGS neurons only fire for large input currents, with a threshold dependent on the level of intrinsic noise; thus, the two neuron types have different firing rates. To evaluate how the networks transmit fluctuations of the slow-varying common input signal, independent of the overall firing rates, we evaluated the averaged population (PSTH) response of each layer, normalized to have a mean equal to 0 and a variance equal to 1 (Figure 4C).

The first few layers of the GS network robustly propagate the slow-varying signal as a result of the temporally jittered response produced by the sensitivity to fast fluctuations at the single neuron level, consistent with the population response in Figure 3F. However, due to the effects of these same noise fluctuations, this population response degrades in deeper layers (Figure 4C, left, see also Figure S1 for 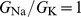). In contrast, the NGS network is insensitive to the fast fluctuations and thresholds the slow-varying input at the first layer, as in Figure 3E. Despite the presence of fast-varying background noise, the NGS network robustly transmits the large peaks of this stimulus to deeper layers without distortion (Figure 4C, right).

This difference in the transmission of information through the two network types is captured in the information between the population response and the slow-varying stimulus in Figure 4D. The GS network initially carries more information about the slow-varying stimulus than the NGS network; however, this information degrades in deeper layers when virtually all the input structure is lost, and drops below the NGS network beyond layer four (Figure 4D, bottom). While the information carried by the NGS network is initially lower than the GS network (due to signal thresholding), this information is preserved across layers and eventually exceeds the GS information.

The observed differences in the propagation of slow-varying inputs between the two network types resemble changes in wave propagation during development. While spontaneous waves cross cortex in stereotyped activity events that simultaneously activate large populations of neurons at birth, these waves disappear after the first postnatal week [13], [16]. We have demonstrated that immature neurons lacking the gain-scaling ability can indeed propagate slow-varying wave-like input of large amplitude as population activity across many layers. As these same neurons acquire the ability to locally scale the gain of their inputs and efficiently encode fast fluctuations, they lose the ability to propagate large amplitude events at the population level, consistent with the disappearance of waves in the second postnatal week [13]. While many parameters regulate the propagation of waves [14], [29], our network models demonstrate that varying the intrinsic properties of single neurons can capture substantial differences in the ability of networks to propagate slow-varying information. Thus, changes in single neuron properties can contribute to both spontaneous wave generation and propagation early in development and the waves' disappearance later in development.

### Dynamics of signal propagation

The layer-by-layer propagation of a slow-varying signal through the population responses of the two networks can be qualitatively predicted using a mean field approach that bridges descriptions of single neuron and network properties. Since network dynamics varies on faster timescales than the correlation timescale of the slow-varying signal, the propagation of a slow-varying signal can be studied by considering how a range of mean inputs propagate through each network. The intrinsic response of the neuron to a mean (DC) current input is quantified by the 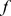
–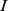 curve which averages over the details of the fast background fluctuations; yet, the magnitude of background noise, 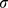, can change the shape and gain of this curve [20], [21]. Thus, for a given neuron type, there is a different 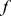
–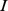 curve depending on the level of noise 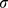, 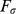 (Figure 1C,D). One can approximate the mean current input to a neuron in a given layer 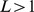, 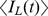, from the firing rate in the previous layer 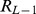 through a linear input-output relationship, with a slope 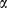 dependent on network properties (connection probability and synaptic strength, see Eq. 15). Given the estimated mean input current for a given neuron in layer 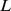, 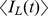, the resulting firing rate of layer 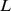, 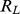, can then be computed by evaluating the appropriate 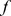
–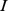 curve, 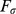, which characterizes the neuron's intrinsic computation
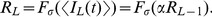
(1)


**Figure 5:**
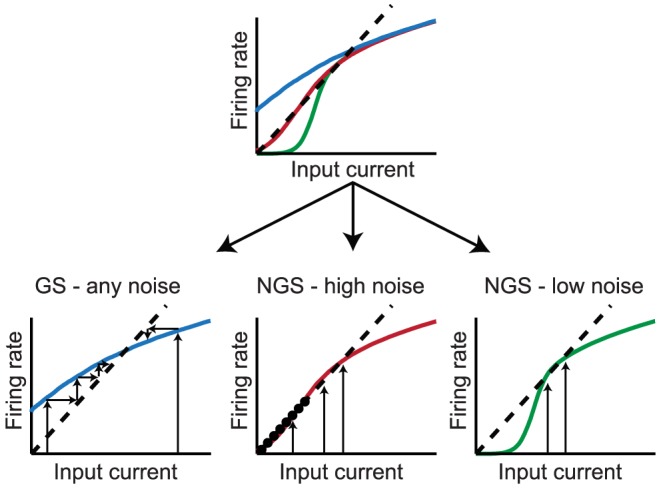
Fixed points of the iterated map dynamics. Top: An illustration of three 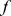
–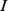 curves (colors) and the corresponding linear input-output relation (black dashed) with slope 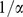 derived from the mean field. Bottom left: The dynamics has a single stable fixed point and all input currents are attracted to it (indicated by small arrows converging to the fixed point). This corresponds to 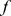
–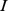 curves of GS neurons at all values of 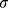. Middle: The dynamics has a line of stable fixed points that allow robust transmission of a large range of input currents in the network. NGS neurons with high values of 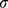 have such dynamics. Right: The stable line of fixed points is smaller for 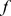
–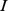 curves that are more "thresholding,'' corresponding to NGS neurons with low 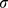.

**Figure 6:**
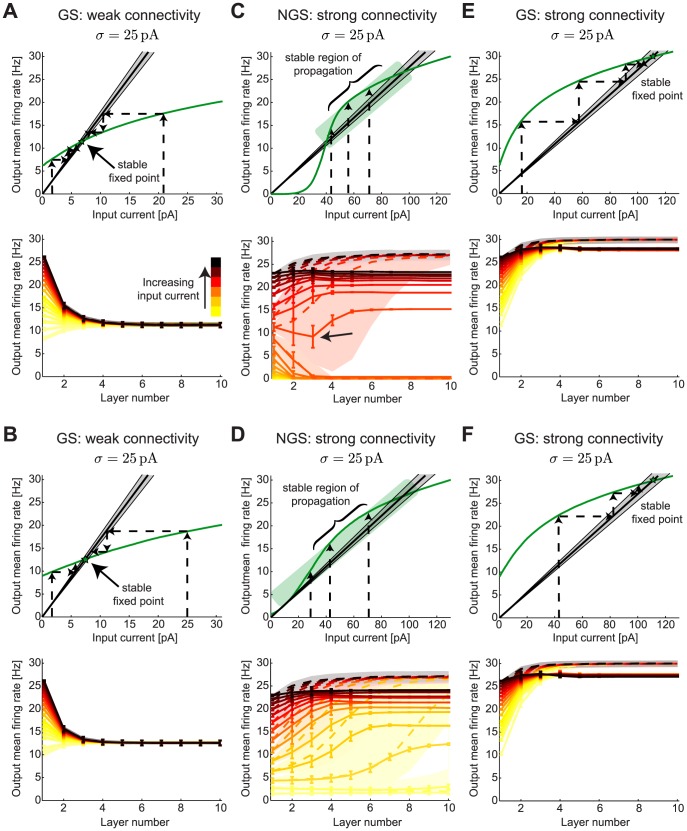
Firing rate propagation through networks of gain-scaling and nongain-scaling neurons. **A,B.** Top: The 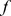
–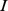 curves (green) for GS neurons (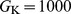 pS/µm^2^ and 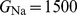 pS/µm^2^) at two levels of noise, 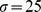 pA (low noise) and 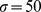 pA (high noise). The linear input-output relationships from the mean field (black) predict how the mean output firing rate of a given network layer can be derived from the mean input current into the first layer with the standard deviation of the prediction shown in gray. Dashed arrows show the iterated map dynamics transforming different mean input currents into a single output firing rate determined by the stable fixed point (green star). Bottom: The network mean firing rates for a range of mean input currents (to layer 1) as a function of layer number, with a clear convergence to the fixed point by layer 5. The results from numerical simulations over 10 second-long trials are shown as full lines (mean 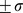 from 2000 neurons in each layer) and mean field predictions are shown in dashed lines with a shaded background in the same color (for each different input) illustrating the standard deviation of the prediction. Other network parameters: connection probability 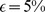, synaptic strength 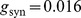 and range of mean input currents 0–22 pA. **C,D.** Same as A,B but for NGS neurons (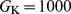 pS/µm^2^ and 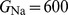 pS/µm^2^) with stronger synaptic strength 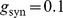 and range of mean input currents 0–70 pA. The network dynamics show a region of stable firing rate propagation (green box) where the 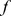
–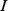 curve behaves like it is tangent to the input-output line for a large range of mean input currents (to layer 1). The size of the region increases with noise (until 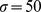 pA). Bottom panels show the transmission of a range of input firing rates across different layers in the network. The arrow denotes a case where the firing rate first decreases towards 0 and then stabilizes. **E,F.** Same synaptic strength as C,D but for GS neurons (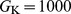 pS/µm^2^ and 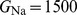 pS/µm^2^). Bottom panels show the convergence of firing rates to a single fixed point similar to the weakly connected GS network in A,B. As for the NGS networks in C,D, the mean field analysis predicts convergence to a slightly higher firing rate than the numerical simulations.

Thus, these two curves serve as an iterated map whereby an estimate of the firing rate in the *L*th layer, 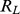, is converted into a mean input current to the next layer, 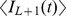, which can be further converted into 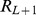, propagating mean activity across multiple layers in the network (Figures 5, 6). While for neurons in the first layer, the selected 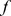
–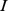 curve is the one corresponding to the level of intrinsic noise injected into the first layer, 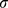, for neurons in deeper layers, the choice of 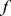
–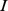 curve depends not only on the magnitude of the independent noise fluctuations injected into each neuron, but also on the fluctuations arising from the input from the previous layer (see Eq. 16 in Methods). The behavior of this iterated map is shaped by its fixed points, the points of intersection of the 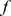
–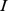 curve 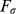 with the input-output line 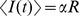, which organize the way in which signals are propagated from layer to layer. The number, location and stability of these fixed points depend on the curvature of 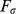 and on 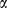 (Figure 5). When the slope of 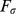 at the fixed point is less than 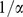, the fixed point is stable. This implies that the entire range of initial DC inputs (into layer 1) will tend to iterate toward the value at the fixed point as the mean current is propagated through downstream layers in the network (Figure 5, left). Therefore, all downstream layers will converge to the same population firing rate that corresponds to the fixed point. In the interesting case that 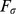 becomes tangent to the linear input-output relation, i.e. the 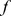
–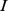 curve has a slope equal to 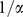, the map exhibits a *line attractor*: there appears an entire line of stable fixed points (Figure 5, middle). This ensures the robust propagation of many input currents and population rates across the network. Interestingly, the 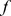
–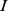 curves of the GS and NGS neurons for different values of 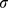 fall into one of the regimes illustrated in Figure 5: GS neurons with their 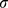-invariant 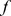
–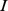 curves have a single stable fixed point (Figure 5, left), while the NGS neurons have line attractors with exact details depending on 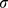 (Figure 5, middle and right). The mechanics of generating a line attractor have been most extensively explored in the context of oculomotor control (where persistent activity has been interpreted as a short-term memory of eye position that keeps the eyes still between saccades) and decision making in primates (where persistent neural activity has been interpreted as the basis of working memory) [30].

Indeed, Figure 6A,B shows that the 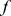
–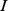 curves for GS neurons at two values of 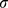, one low and one high, are very similar. The mean field analysis predicts that all initial DC inputs applied to layer 1 will converge to the same stable fixed point during propagation to downstream layers. Numerical simulations corroborate these predictions (Figure 6A,B, bottom). A combination of single neuron and network properties determine the steady state firing rate through 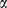 (Eq. 15). Activity in the GS networks can propagate from one layer onto the next with relatively weak synaptic strength even when the networks are sparsely connected (5% connection probability), as a result of the low thresholds of these neurons (Figure 1D). The specific synaptic strength in Figure 6A,B was chosen arbitrarily so that the 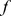
–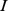 curve intersects the input-output line with slope 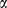, but choosing different synaptic strength produces qualitatively similar network behavior (Figure S2). The parameter 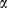 can be modulated by changing either the connectivity probability or the synaptic strength in the network; as long as their product is preserved, 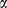 remains constant and the resulting network dynamics does not change (Figure S2). Furthermore, as a result of the lack of modulability of GS 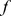
–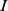 curves by 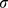 (Figure 1D), the network dynamics remains largely invariant to the amplitude of background noise.

In contrast, the amplitude of background noise fluctuations, 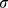, has a much larger impact on the shape of NGS 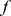
–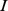 curves (Figure 1C) and on the resulting network dynamics (Figure 5). When the combination of sparse connection probability and weak synaptic strength leads to the slope 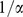 being too steep (weak connectivity in GS networks, Figure 6A,B), there may be no point of intersection with the NGS 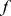
–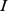 curves: all DC inputs are mapped below threshold and activity does not propagate to downstream layers. Keeping the same sparse connection probability of 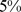 and increasing synaptic strength enables the propagation of neuronal activity initiated in the first layer to subsequent layers in NGS networks. For a particular value of 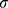, there is an entire line of stable fixed points in the network dynamics (Figure 5, middle), so that a large range of input currents are robustly transmitted through the network. More commonly, however, the map has three fixed points: stable fixed points at a high value and at zero, and an intermediate unstable fixed point (Figure 6C,D). In this case, mean field theory predicts that DC inputs above the unstable fixed point should flow toward the high value, while inputs below it should iterate toward zero, causing the network to stop firing. However, the map still behaves as though the 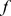
–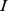 curve and the input-output transformation are effectively tangent to one another over a wide range of input rates (green box in Figure 6C,D), creating an effective line of fixed points for which a large range of DC inputs is stably propagated through the network; this is generically true for a wide range of noise values, although the exact region of stable propagation depends on the value of 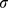 (Figure 5, middle and right, Figure S3). The best input signal transmission is observed when the network noise selects the *most linear* 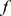
–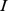 curve that simultaneously maximizes the range of DC inputs and population firing rates of the neurons (Figure 5, middle). This is approximately the noise value selected in Figure 6C,D. We call this a *stable region of propagation* for the network since a large range of mean DC inputs can be propagated across the network layers so that the population firing rates at each layer remain distinct. Our results resemble those of van Rossum et al. [31] where regimes of stable signal propagation were observed in networks of integrate-and-fire neurons by varying the DC input and an additional background noise. The best regime for stable signal propagation occurred for additive noise that was large enough to ensure that the population of neurons independently estimated the stimulus, as in our NGS networks (Figure 5, middle and right, Figure S3).

The emergence of extended regions of stable rate propagation implies that the NGS mean field predictions (Figure 6C,D, bottom) are less accurate than for the GS networks where the convergence to the stable fixed points is exact (Figure 6A,B). However, the NGS mean field predictions show qualitative agreement with the simulation results, in particular in the initial network layers where the approach to the nonzero stable fixed point is much slower than in the GS networks, i.e. occurs over a larger number of layers. Along with the slow convergence of firing rates toward a single population firing rate, the ability of network noise to modulate the NGS 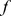
–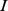 curves suggests that multiple 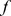
–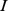 curves can be used to predict network dynamics by combining added and intrinsically generated noise (see Eq. 16). As a result, for some input currents (e.g. arrow in Figure 6C) the firing rate goes down in the first three layers where network dynamics predicts convergence to the zero stable fixed point. The initial decrease of firing rate is due to the disappearance of weak synaptic inputs that cannot trigger the cells to spike. Network noise then selects a different 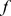
–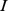 curve that shifts the dynamics into the rate stabilization region (Figure 6C, green box) where firing rates are stably propagated. The onset of synchronous firing of the neuronal population in each layer also contributes to rate stabilization. Population firing rates in deeper layers increase to a saturating value lower than the mean field predicted value. Similar results have been observed experimentally [32] and in networks of Hodgkin-Huxley neurons [33]. We find similar network dynamics for a more weakly connected NGS network using the smallest possible synaptic strength that allows activity to propagate through the network (Figure S2). As for the GS networks, as long as the product of connection probability and synaptic strength is constant, the slope of the input-output linear relationship 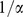, and the network dynamics remain unchanged, even if these network parameters change individually (Figure S2).

An exception to this result is observed at very sparse connectivity (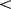
2%), where network behavior is more similar to the GS networks (Figure S2, bottom right). At this sparse connectivity, independent noise reduces the common input across different neurons and synchrony is less pronounced. This argues that the emergence of synchrony plays a fundamental role in achieving reliable propagation of a range of DC inputs (and correspondingly population firing rates) in the NGS networks. Although experimental measurements of the connectivity probability in developing cortical networks are lacking, calcium imaging of single neurons demonstrates that activity across many neurons during wave propagation is synchronous [34]. Intracellular recordings of adult cultured cortical networks also demonstrate that synchronous neuronal firing activity is transmitted in multiple layers [32].

To examine network behavior for comparable connectivity strength, we repeated the network simulations and mean field predictions of mean DC input propagation in GS networks with the same increased synaptic strength needed for propagation of activity in the NGS networks. We found that the behavior was similar to the weakly connected GS network: Regardless of the initial input current, the network output converged to a single output firing rate by layer 5 (Figure 6E,F), making these networks incapable of robustly propagating slow-varying signals without distortion. As for the strongly connected NGS networks, neurons across the different layers in these strongly connected GS networks developed synchronous firing. This synchrony led to a small difference (several Hz) between the final firing rate approached by each network compared with the firing rate predicted from the mean field analysis. Although both the strongly connected GS and NGS networks developed synchronous firing, the behavior of the two types of networks remained different (Figure 6).

The results in this section indicate that firing rate transmission depends on the details of single neuron properties, including their sensitivity to fast fluctuations as characterized by the LN models (Figure 1A,B). Firing rate transmission also depends on the modulability of the 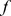
–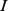 curves by the noise amplitude 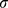 (Figure 1C,D). Because of these differences in intrinsic computation, the GS and NGS networks show distinct patterns of information transmission (Figure 5): firing rate convergence to a unique fixed point, or a line of fixed points ensuring stable propagation of firing rates which can be reliably distinguished at the output, respectively. In the latter case, even when a line of fixed point is not precisely realized as in Figure 5 (middle), competition between the slow convergence of firing rates to the mean field fixed point and the emergence of synchrony enable the propagation of firing rates through the different network layers, aided by the range of 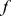
–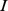 curves sampled by network noise with amplitude 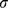.

### Implications of single unit computational properties for information transmission

**Figure 7:**
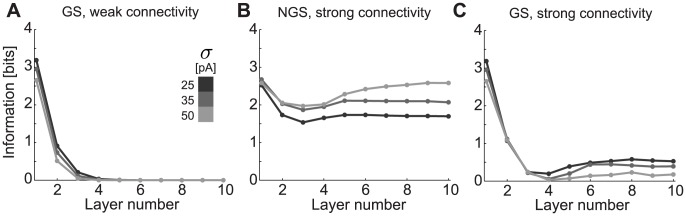
Mutual information about the mean stimulus transmitted by GS and NGS networks. The mutual information as function of layer number for **A**. weakly connected GS (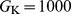 pS/µm^2^ and 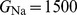 pS/µm^2^), **B**. strongly connected NGS (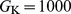 pS/µm^2^ and 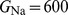 pS/µm^2^) and **C**. strongly connected GS networks (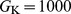 pS/µm^2^ and 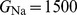 pS/µm^2^) as shown in Figure 6 for different noise levels indicated by the shade of gray.

Given the predicted signal propagation dynamics, we now directly compute the mutual information between the mean DC input injected into layer 1 and the population firing rates at a given layer for each magnitude of the independent noise 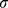 (Figure 7). This measures how distinguishable network firing rate outputs at each layer are for different initial mean inputs. The convergence of population firing rates across layers to a single value in the GS networks leads to a drop in information towards zero for both the weakly (Figure 6A,B) and strongly connected GS networks (Figure 6E,F) as a function of layer number and for a wide range of network noise 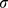 (Figure 7A,C). NGS networks can transmit a range of mean DC inputs without distortion (Figure 6C,D); thus, the information between input DC and population firing rate remains relatively constant in subsequent layers (Figure 7B). The information slightly increases in deeper layers due to the emergence of synchronization, which locks the network output into a specific distribution of population firing rates. As noise amplitude increases, the selected 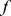
–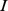 curve becomes tangent to the linear input-output relationship over a larger range of input firing rates (Figure 6C,D); hence, a larger range of inputs is stably transmitted across network layers. Counterintuitively, this suggests that increasing noise in the NGS networks can serve to increase the information such networks carry about a distribution of mean inputs.

### Origins of firing rate modulability by noise magnitude

**Figure 8:**
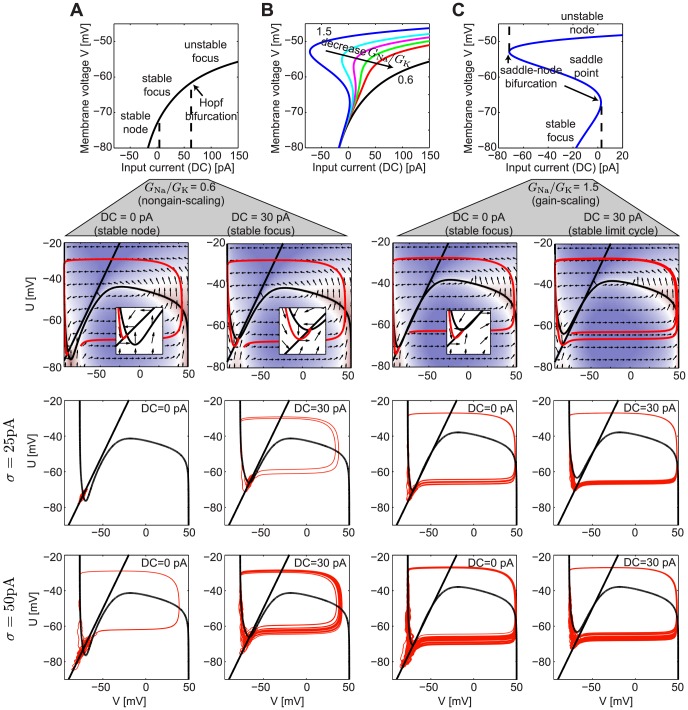
Analysis of the reduced Mainen model. **A**. Top: Fixed points and their stability for the dynamics of a NGS neuron with 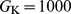 pS/µm^2^ and 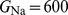 pS/µm^2^ (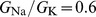) as a function of the input current DC. Bottom: The phase planes showing the nullclines (black) and their intersection points (fixed points) together with the flow lines indicated by the arrows. A single trajectory is shown in red. The inset shows a zoomed portion of the phase plane near the fixed point. Below we show trajectories for two values of 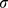 and two DC values. **B**. The fixed points for different ratios 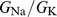, while keeping 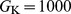 pS/µm^2^ and varying 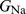, as a function of the DC. **C**. Same as A but for a GS neuron with 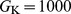 pS/µm^2^ and 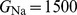 pS/µm^2^ (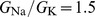). Note that the abscissa has been scaled from A and B.

The differential ability of GS and NGS networks to reliably propagate mean input signals is predicted by the modulability of the 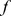
–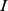 curves by the network noise 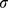. To understand the dynamical origins of this difference, we analytically reduced the neuron model (Eq. 2) to a system of two first order differential equations describing the dynamics of the membrane potential 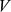 and an auxiliary slower-varying potential variable 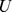 (Methods) [35]. We analyzed the dynamics in the phase plane by plotting 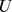 vs. 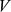. The *nullclines*, curves along which the change in either 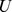 or 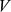 is 0, organize the flows of 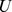 and 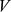 (Figure 8); these lines intersect at the fixed points of the neuron's dynamics. We studied the fixed points at different ratios of 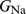 and 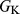, with a particular focus on the values discussed above (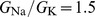 and 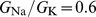). These exhibit substantial differences in the type and stability of the fixed points, as well as the emergent bifurcations where the fixed points change stability as one varies the mean DC input current into the neuron (Figure 8).

For a large range of DC inputs, the NGS neuron (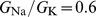) has a single stable fixed point (either a node or a focus) (Figure 8A). In this case, the only perturbation that can trigger the system to fire an action potential is a large-amplitude noise current fluctuation. The 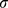 of the current then determines the number of action potentials that will be fired in a given trial and strongly modulates the firing rate of the neuron. We show two trajectories at 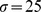 pA and 50 pA and at two different DC values of 0 and 30 pA (Figure 8A), at which the 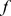
–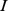 curves are strongly noise-modulated (Figure 1C). As the DC increases beyond 62 pA, the fixed point becomes unstable and a stable limit cycle emerges (not shown). In this case, any 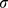 will move the trajectories into the stable limit cycle and the neuron will continuously generate action potentials, with a firing rate independent of 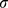. Indeed, Figure 1C shows that the 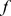
–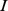 curves become less effectively modulated by 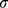 for DC values greater than 62 pA.

As the conductance ratio 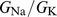 increases, the range of DC values for which the system has a single fixed point decreases (Figure 8B). Indeed, the GS neuron (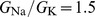) has a stable limit cycle for the majority of DC values (Figure 8C). This implies that GS neurons are reliably driven to fire action potentials for any 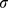 and their firing rate is not very sensitive to 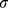. For low DC values, the stable limit cycle coexists with a stable fixed point, so in this case 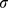 of the noise can modulate the firing rate more effectively, as is seen in Figure 1D.

This analysis highlights the origins for the differential modulability of firing rate in NGS and GS neurons. Although the model reduction sacrifices some of the accuracy of the original model, it retains the essential features of action potential generation: the sudden rise of the action potential which turns on a positive inward sodium current, and its termination by a slower decrease in membrane potential which shuts off the sodium current and initiates a positive outward potassium current hyperpolarizing the cell. Although simpler neuron models (e.g. binary and integrate-and-fire [36]–[38]) allow simple changes in firing thresholds, the dynamical features inherent in the conductance-based neurons studied here are needed to capture noise-dependent modulation.

## Discussion

The adult brain exhibits a diversity of cell types with a range of biophysical properties. Organized into intricate circuits, these cell types contribute to network computation, but the role of intrinsic properties is unclear. Recently, we have shown that during early development, single cortical neurons acquire the ability to represent fast-fluctuating inputs despite variability in input amplitudes by scaling the gain of their responses relative to the scale of the inputs they encounter [8]. Before these intrinsic properties shift, the developing cortex generates and propagates spontaneous waves of large-scale activity [13], [22], [39], [40], which regulate developmental changes in ion channel expression, synaptic growth and synaptic refinement processes [29], [41], [42]. How do experimentally observed biophysical properties affect ongoing network dynamics at this time? Using model neurons with conductance properties chosen to reproduce this developmental change in gain scaling, we investigated the implications of this change on the ability of feedforward networks to robustly transmit slow-varying wave-like signals. The conductance-based models that we considered are not intended as an exact biophysical model for developing cortical neurons; rather they allow us to study the more fundamental question of the role of single neuron computation on network behavior in a case with a well-defined and physiologically relevant network level property.

We add to previous studies by considering first, the fidelity of propagation of *temporally varying* patterns by biophysically realistic neurons, basing our work in a biological context where the brain naturally enters a state of wave propagation. Second, our work highlights a role of cellular processes in large-scale network behavior that has rarely been studied. Our results implicate intrinsic conductance change as a way to switch between global synchronization and local responsiveness, rather than synaptic plasticity, which is typically used to evoke such a global network change [17]. Related changes in excitability that accompany the cessation of spontaneous activity have been observed in the mouse embryonic hindbrain, where they have been ascribed to hyperpolarization of resting membrane potential and increased resting conductance of 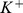 channels [43]. Finally, we analyze network information transmission on two different timescales (local fluctuations and network-wide wave-like events) and thereby generalize previous classification of feedforward network propagation into either *synchrony-based coding* [32], [44], and *rate-based coding* [31], [45].

We use two different descriptions of neuronal properties to characterize the neuron's ability to propagate information at these different time- and lengthscales. The processing of fast input fluctuations can be characterized using LN models [8], [46]–[48]. While single neuron properties affect the linear feature [46], [48], [49], here we focus on the scaling of the nonlinearity in the LN model to stimuli of different amplitudes. Information about slowly modulated input is described using noise-modulated 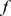
–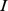 curves [20], [21], [50]. This ability of developing neurons to transmit distinct information at two different timescales is an example of a *temporally multiplexed* code [3], [51]–[53]. Here, GS neurons perform temporal multiplexing as they simultaneously convey distinct information about fast and slow fluctuations, reliably encoding slowly varying stimuli, albeit only for a few network layers. The NGS neurons also implement a multiplexed code because of their dual role to transmit firing rates while maintaining synchrony.

The above characterizations predict the success of global information propagation across multiple network layers [49], [50]. In integrate-and-fire network models with a *fixed* 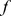
–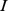 curve, different network dynamics has been achieved by varying connectivity probability and synaptic strength [31], [45], [54], [55]. Here, in addition we considered the modulation of the 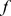
–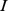 curves by the combined effects of injected independent noise and measured correlated noise from network interactions, permitting a description of network responses dependent on the input statistics, intrinsic single neuron properties and network connectivity (Figure 6). The role of 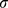-modulated 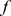
–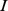 curves has also been fundamental in understanding how intrinsic neuron properties affect correlation transfer and encoding of rate- and synchrony-based signals in reduced networks of two neurons stimulated with a common input signal and independent noise [48], [49], [52], [53], [56]. We expect that generalizations of these methods will enable improved theoretical predictions for firing rate and correlation transfer beyond mean field, by computing the effects of temporal correlations such as we observe.

Firing rate transmission in our NGS networks co-occurs with the development of precise spike-time synchronization over a wide range of stimulus statistics and network connectivity (Figure 6). This synchronization might be a feature of biologically inspired networks because similar patterns were reported in experimentally simulated feedforward networks *in vitro* [32] and Hodgkin-Huxley-based simulations [33], but not in networks of threshold binary neurons [36], [57], nor integrate-and-fire neurons [55]. Several manipulations to single neuron or network properties might reduce this synchrony. These include: introducing sparse connectivity with strong synapses [17], [37], increasing independent noise input [31], [36], or embedding the feedforward into recurrent networks with inhibition to generate asynchronous background activity [37], [38], [55], [58]; but these typically result in signal degradation or implausible assumptions in our models. We did not find a regime supporting reliable asynchronous rate propagation, consistent with other studies [32], [33], [36], [44].

We identified the biophysical basis of the single-unit properties that underlies our results. The change in gain scaling is accompanied by a difference in the distance from rest to threshold membrane potential [8]: GS neurons have a smaller distance to threshold and are more likely to fire driven by noise fluctuations, while NGS neurons have a larger distance to threshold and must integrate many coincident inputs to fire. Indeed, a change in spiking threshold in simpler model neurons has been shown to modulate the mode of signal transmission in a feedforward network [36], [59], [60]. However, our mean-field and phase-plane dynamical analyses together show that threshold is not the only factor at work: the nature of rate propagation is intimately connected with the bifurcation properties of the neuron model. While we focused on two representative contrasting cases, these properties vary systematically with the conductance ratio of the neuron and we have mapped out the spectrum of possible behaviors of this model.

The robustness of information propagation across network layers is likely to have important implications for how developmental information contained in wave propagation patterns is transmitted across the cortex. We have previously shown that cortical waves are initiated in a pacemaker circuit contained within the piriform cortex [12]–[14], which is likely to provide the strong input necessary to drive NGS neurons. The waves propagate dorsally across the neocortex so that throughout the developmental period of wave generation, the neocortex acts as a follower region in the sequence of wave propagation. The reliability with which firing patterns of piriform neurons are retained as waves propagate into the neocortex will determine the nature of developmental information that the neocortex receives from those waves during its development. As gain scaling develops, more mature neurons can support efficient coding of local fluctuations and discard information about network-wide events. Therefore, the alteration of a single developmentally regulated conductance parameter can shift cortical neurons from synchrony-based encoders of slow inputs to noise-sensitive units that respond with high fidelity to local fluctuations independent of the overall scale. The growing sensitivity to noise of cortical neurons in the first postnatal week might help to prevent large-scale wave activity from dominating adult neural circuits, thus discouraging epileptiform patterns of network activity. At the same time, the emergence of gain scaling supports a transition to a state in which cortical circuits, rather than participating in network-wide events, can respond optimally to appropriately scaled local information, breaking up the cortical sheet into smaller information-processing units.

The mature cortex is also capable of generating spontaneous activity that propagates over large distances in the absence of sensory stimulation [61]–[63]. Such wave activity is postulated to be involved in short-term memory and the consolidation of recent transient sensory experience into long-lasting cortical modifications. For example, recent *in vivo* experiments proposed that synaptic plasticity is enforced by slow waves that occur during sleep [64, 65]. Spontaneous propagation activity patterns emerge from the interplay of intrinsic cellular conductances and local circuit properties [63]; our results raise the possibility that modulation of intrinsic properties through slow Na^+^ inactivation or neuromodulation could have multiple short-term effects on cortical information processing.

While we have examined the effect of gain scaling as a specific form of adaptation emerging during development, other adaptation mechanisms also likely play an important role in information transmission in feedforward networks. For instance, spike frequency adaptation has been shown to have effects that accumulate across multiple layers of feed-forward networks [31]. This widely observed form of adaptation can arise from calcium-dependent potassium conductances which generate AHPs [21], [66], [67]. Indeed, we and others have found that AHP-generating conductances can also support gain scaling behavior by single neurons [9], [68]. Independent of AHP conductances, slow sodium channel inactivation can also contribute to spike frequency adaptation [69], [70]. Incorporating such slow-timescale channel dynamics will require taking into account temporal aspects of the coding of mean (or variance) [71] that are presently ignored in our mean-field analysis based on modulated 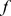
–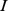 curves. These slow dynamics may contribute to successive layers of filtering that affect information transmission [10]. An analytical characterization of the impact of slow neuronal dynamics on networks is likely to require novel theoretical approaches beyond those used here.

Similarly, other factors beyond the specific changing intrinsic neuronal properties addressed here contribute to the generation of spontaneous cortical waves with complex spatio-temporal properties. During the same developmental time period, the cortex undergoes substantial changes in information processing capacity that are beyond the scope of the present study [72]–[74]. Activity-dependent modification of synaptic connections driven by developmental cues contained in spontaneous wave patterns are likely to refine cortical networks into their mature state [14], [16], [39], [42], [73]. Furthermore, the emergence of synaptic inhibition as GABA becomes more hyperpolarizing contributes to diminishing the wave-like activity generated by the immature excitatory network [14], [73]. Thus, synaptic plasticity and intrinsic neuronal properties interact to modulate the emergence, propagation and the eventual disappearance of spontaneous waves in the developing cortex, and also to endow spatially-distinct regions at different time points with different information processing capabilities.

## Materials and Methods

### Single neuronal models

We studied a modified version of a Hodgkin-Huxley style model adapted by Mainen *et al.* [75] for spike initiation in neocortical pyramidal neurons. The model consists of a leak current, mammalian voltage-gated transient sodium and delayed-rectified potassium currents with maximal conductances 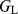, 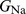 and 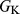, and reversal potentials 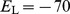 mV, 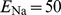 mV and 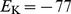 mV:
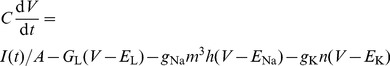
(2)


where 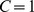 *µ*F/cm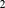 is the specific membrane capacitance and 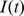 is the input current with 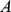 denoting the area of the membrane patch with radius of 30 *µ*m. The leak conductance was set to 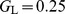 pS/*µ*m^2^ such that the membrane time constant at the resting potential was 40 ms (any values between 25 and 50 ms were consistent with experimental data) [8]. The active conductances can be expressed via the gating variables 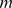, 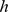 and 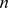 such that 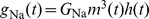 and 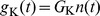. We used 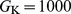 pS/*µ*m^2^ and 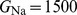 pS/*µ*m^2^ for the maximal conductances of the GS neurons, so that their ratio was 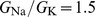; and 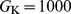 pS/*µ*m^2^ and 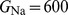 pS/*µ*m^2^ for the maximal conductances of the NGS neurons, so that their ratio was 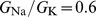. We also studied a larger range of these maximal conductances in Figure 2. The gating variables have the following kinetics: 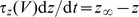 with 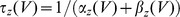 where 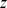 can be 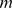, 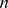 or 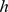, and:
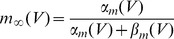
(3)

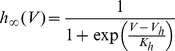
(4)

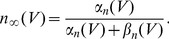
(5)


The rate coefficients, 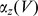 and 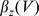 are of the form 
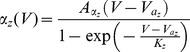
 and 
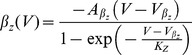
 and the kinematic parameters are provided in Table 1.

**Table 1:** Kinetic parameters of the biophysical model.

variable	equation	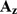 [ 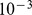 ]	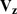 [mV]	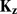 [mV]
m	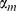	182	−35	9
	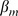	124	−35	9
h	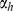	24	−50	5
	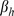	9.1	−75	5
	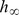	–	−65	6.2
n	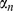	20	20	9
	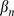	2	20	9

The equations were numerically solved using a first-order Euler method with an integration time step of 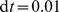 ms. We used a threshold of −20 mV to detect spikes, although our results did not depend on the exact value of this parameter.

### Fitting linear-nonlinear models

For spike-triggered characterization we injected Gaussian noise current, 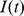, with mean, 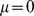, and standard deviation, 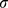, to elicit spike trains in ten 1000-second long trials. All input current traces were realizations of the Ornstein-Uhlenbeck process [76] expressed as:
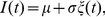
(6)


where 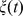 has unit variance and correlation time of 1 ms to match experimental conditions [8].

Intrinsic computation in these neuron types was previously characterized in experiments and model neurons [8] using a one-dimensional Linear-Nonlinear (LN) cascade model of output spike times to the input Gaussian current stimulus with standard deviation 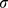 [23]. The first component of the LN model is a feature which linearly filters the stimulus producing the amplitude of the feature present in the input; the second component is a nonlinear function which gives the instantaneous firing rate for each value of the filtered stimulus. We take the feature to be the spike-triggered average (STA) [18], [24], and obtain the expression for the nonlinear response function from Bayes' law:
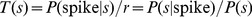
(7)


where 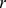 is the mean firing rates for fixed input mean and standard deviation 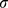, 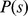 is the prior distribution which is a Gaussian with mean zero and variance 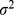, 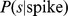 is the spike-triggered stimulus distribution obtained from the histogram of filtered stimulus values when the spikes occur.

We refer to the neurons with 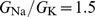 ratio equal to 1.5 as *gain-scaling*, because scaling the stimulus by 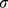 produces a nonlinearity in the LN model that is independent of 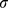, *i.e.* 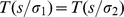 for inputs with two different standard deviations 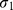 and 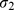 (mean fixed to zero in Figure 1A,B, red) [8]. The neurons with 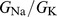 ratio equal to 0.6 are termed *nongain-scaling*, because nonlinearities in the LN model vary with different values of the standard deviation when the stimulus is scaled by 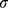 (Figure 1A,B, blue). The gain-scaling properties of single neurons hold for all 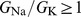 [8].

### Network dynamics

We considered a feedforward network architecture with 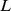 layers, each layer consisting of 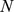 neurons (Figure 4A). We considered networks of 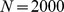 neurons (the results remain the same as long as 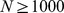). A common temporally fluctuating input current was injected to all neurons in the first layer. The common input was generated using
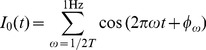
(8)


where 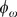 is a random phase in 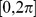, and 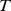 is the total length of the stimulus. The exact properties of this stimulus (size of the window 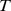, the cutoff frequency of 1 Hz) were not important, as long as the correlation timescale of this stimulus was much longer than the correlation timescale of the fast fluctuations (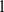 ms) independently injected into each neuron.

Instead of 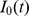, neurons in deeper layers (beyond the first) received synaptic input from neurons in the previous layer via conductance-based synapses. In contrast to current-based synapses, conductance-based synapses have been shown to support the stable propagation of synfire chains [38] and a larger range of firing rates [37]. The synaptic input current into a neuron in layer 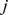 in the network (which receives inputs from a subset of neurons in the previous layers) is given by
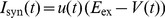
(9)


where 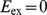 mV is the excitatory reversal potential and 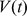 is the membrane potential of the neuron. The synaptic conductance 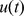 is a continuous variable which increases with the spike times of each input 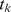 by the excitatory postsynaptic potential (EPSP) scaled by the corresponding synaptic strength 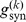. We used exponentially decaying EPSPs with a time constant 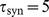 ms. Then we can write the synaptic conductance as
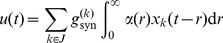
(10)


where 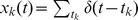 is the delta spike train of the 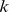-th neuron in the previous layer with spikes at times 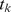 and 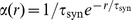 when 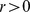 is the EPSP. 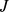 denotes a random subset of the 2000 neurons in the previous layer providing synaptic input into the given neuron.

There were no recurrent connections among the neurons. Each neuron in the network also received an independent noise input with mean 0 and standard deviation 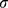 that fluctuates on a timescale significantly shorter than the timescale of the common input to represent random synaptic input that cortical networks experience during early development [28]. In all models, the noise stimulus added to each neuron was independent from the mean stimulus and correlated with a correlation time of 1 ms. Note that for the mean field analysis (see below), simulations were performed with a constant mean 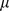 (Figure 6), rather than the time-dependent 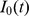 (Equation 8). The range of stimulus standard deviations 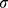 was chosen to produce firing rates larger than 3 Hz and such that voltages were not hyperpolarized below 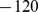 mV to match the corresponding experiments [8].

### Mean field analysis

Given an input current 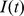, the output firing rate can be expressed by the 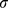-dependent 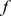
–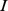 curve: 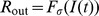. We computed the 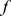
–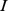 curves for the GS and NGS neurons for a range of mean inputs 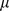 and fluctuation amplitudes 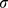 (Figure 1C,D) from 100 second long simulations. The mean current ranged from 0 to 120 pA in steps of 2.5 pA and the standard deviation from 5 to 150 pA in steps of 2.5 pA.

The mean field analysis was used to predict firing rate transmission across the network (Figure 6). Given the synaptic current into a neuron in layer 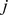 in the network (which receives inputs from a subset of neurons in the previous layers connected with weights of strengths 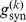), the average synaptic current received by a neuron in one layer from a subset (or all) of neurons in the previous layer can be written as:
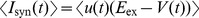
(11)


where the angle brackets denote average over time. In the limit that 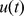 and 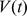 are uncorrelated, then
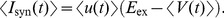
(12)


The average synaptic conductance can be written as
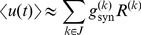
(13)


where 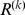 is the average firing rate of neuron 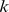. We let 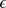 denote the connection probability between neurons in two consecutive layers; therefore, the subset 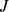 has approximately 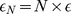 neurons. We examined connectivity probability ranging between 0.5%, 5% and 10% while keeping the product of the connectivity probability 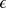 and synaptic strength 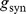 fixed, and observed no differences in how effectively firing rates were propagated across different layers in the network (Figure S2). The main results use 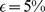. For the two network types, we chose synaptic strength sufficiently strong to allow for activity to be maintained in each network. For the NGS network we used 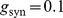, while for the GS network we explored in addition weaker synaptic strength of 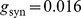; although the exact values used were not too important as long as the iterated map dynamics predicting the mean firing rates across the network had the same structure (for example, number of fixed points) (Figure S2). Since all synapses in our network are identical to 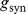, we can approximate 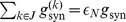; similarly, all the neurons in a given layer are identical so 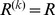. Then the average synaptic current into a neuron in a given layer can be approximated as
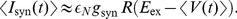
(14)


From the 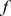
–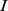 relationship, the firing rate 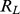 in layer 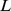 can be expressed as a function of the firing rate of the neurons in the previous layer 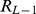 (see Eq. 1) where the scaling coefficient is given by
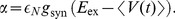
(15)


When computing 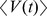 we used only subthreshold voltage fluctuations.

The input-output relationship plotted in Figure 6 (black line) corresponds to the line of slope 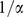. We also computed the standard deviation of the subthreshold voltage fluctuations 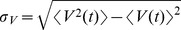 and thus estimated 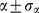 where 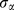 was obtained using Equation 15 with 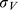 instead of 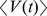. Figure 6 text shows this as a gray boundary around the line with slope 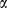, which was used further to interpret the variability of propagation of firing rates.

Furthermore, we note that when predicting the propagation of firing rates across subsequent layers in this mean field analysis, the 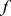
–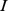 curve 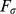 in Equation 1 was chosen such that 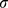 was obtained by combining the standard deviation of the independent noise fluctuations added in each layer 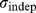, and the standard deviation of the synaptic current recorded in each layer 
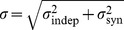
, where
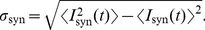
(16)


### Information transmission

We first measured information transmission in the network about slow variations in the input (Figure 4). The mutual information of stimulus and response was computed by testing a particular encoding model (Figure 4D). Typically, this method assumes a model for estimating the stimulus and provides a lower bound on the information transfer because the model does not capture all aspects of the information [77]. We chose the stimulus reconstruction to be a simple population average of the neuronal response (the PSTH), so that the stimulus estimate in layer 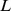, 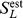, is given by the mean neuronal response obtained from many repetitions of the identical slow stimulus, but different realizations of the fast fluctuations. We computed the information in the 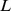-th layer using the equation for a dynamic Gaussian channel [24]

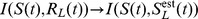


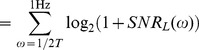
(17)


where the signal-to-noise ratio can be written as
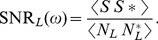
(18)


Assuming Gaussian probability distributions, the noise is
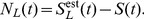
(19)


This quantity computes the information between stimulus and response by taking into account how similar the response (reconstructed stimulus) is to the original stimulus. Due to the different firing rates evoked in the different networks, when computing the information we normalized the reconstructed stimulus (the PSTH) to have zero mean and unit variance.

To quantify the information about fast fluctuations as a function of the mean and 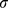 of the input current injected into single neurons (Figure 1E), we used the output entropy of the predicted firing rate probability in the LN model, 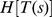, using the nonlinear response function expression from Equation 7.

When examining the fidelity of firing rate transfer in networks composed of the two neuron types, we wanted a measure of how distinguishable is a discrete set of output firing rates in each layer given a set of input currents in the first layer (see Figure 7, note that Figure 1F is like the data in Figure 7 layer 1). This was the information conveyed by the network response of each layer about a stationary mean input 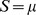, in the presence of background noise 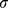 (Figure 7). We obtained the firing rate response of strongly connected NGS and GS networks (synaptic strength 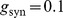) and weakly connected GS networks (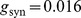) for different layers, noise conditions and ranges of input. For the strongly connected NGS and GS networks, we used a range of 28 input currents uniformly distributed between 0 and 70 pA, and for the weakly connected GS networks, the same number of input currents uniformly distributed in the range of 0 to 22 pA. The noise values that we examined spanned the range of 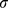 from 15 to 75 pA–which produced biologically relevant output firing rates and subthreshold voltage fluctuations in a valid regime 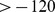 mV. The output firing rates were obtained using 2 second long bins (total length of the trial was 20,000 seconds). Qualitative trends in the information curves were maintained for 1, 5 and 10 second long bins. Then, given the set of firing rate responses 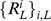 of the neurons of the 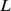-th layer for the 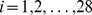 input currents, we constructed 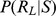 by computing histograms of the output firing rates binned into the same 28 bins. We computed the mutual information for each layer
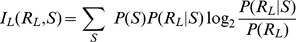
(20)


where 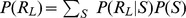 is the probability distribution of the output firing rates [77]. 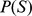 denotes the prior probability of input stimuli which we took to be a uniform distribution so that each stimulus had the same probability 1/28 of occurrence. Although the exact value of the information will depend on the binning choice (here into 28 bins), the contrast in performance of the GS and NGS neurons (which was our goal) was preserved for other binning choices.

### Dynamical systems analysis

To reduce the full conductance-based model (Eq. 2) that depends on four variables, 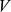, 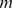, 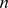 and 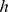, to a system of two first-order differential equations, we followed the procedure described by Abbott and Kepler [35] for the Hodgkin-Huxley model. Although the neuron's membrane potential 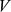 is affected by the three dynamic variables, 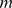, 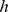 and 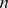, these three do not directly couple to each other but only interact through 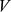. This property allows us to approximate their dynamics by introducing an auxiliary potential variable. Since the time constant that governs the behavior for 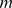 is much smaller than the time constants for 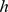 and 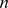, then 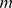 will reach its asymptotic value 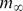 more rapidly than other changes in the model. Therefore, we lose some accuracy in the generation of spikes, but can write 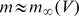. Because of their longer time constants, 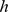 and 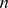 lag behind 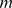 and reach their asymptotic values more slowly. This can be implemented by introducing an auxiliary voltage variable 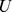 and then replacing 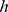 and 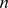 by 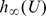 and 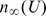, since the functions 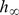 and 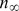 are well separated as a function of the dependent variable, in this case 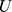. To choose 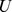, we ask for the time dependence of 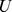 in 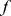 and the time dependence that the slowly changing 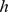 and 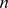 induce into 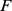 in the full model to match – this is achieved by equating the time derivatives of 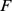 at constant 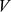 in the full and reduced models. Hence, we convert the full model (Eq. 2) into the following system of first-order differential equations:
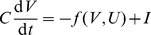
(21)

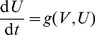
(22)


where
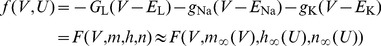
(23)


and 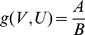 where
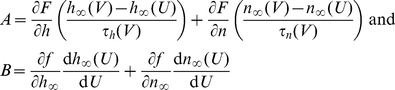
(24)


where 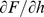 and 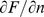 are evaluated at 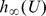 and 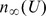.

To study the dynamics of this system in Figure 8, we plotted the nullclines, i.e. the curves where 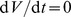 and 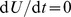. The points where these two curves intersect are the fixed points of the two-dimensional dynamics. In Figure 8 we use arrows in the phase planes to denote the flows around the nullclines.

## Supporting Information

Figure S1
**Information transmission through networks with gain-scaling neurons 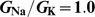 (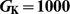 pS/*µ*m^2^ and 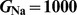 pS/*µ*m^2^).** A. PSTHs from each layer in two networks with different connectivity: left, weak synaptic strength 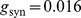; right, stronger synaptic strength 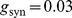. The PSTHs demonstrate that the propagation of a slow-varying input in the presence of a background of fast fluctuations degrades in deeper layers, similar to the gain-scaling networks in Figure 6 of the main text, where 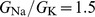. PSTHs were normalized to mean 0 and standard deviation 1 so that the dashed lines next to each PSTH denote 0 and the scalebar 2 normalized units. B. Spike rasters for the PSTHs in A. C. Information about the slow stimulus fluctuations conveyed by the population mean responses shown in A, compare to Figure 4D of the main text.(EPS)

Figure S2
**Firing rate transmission for different connection probability and synaptic strength.** Top: The 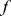
–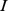 curves (green) for gain-scaling network with 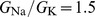 (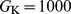 pS/*µ*m^2^ and 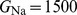 pS/*µ*m^2^) and nongain-scaling networks with 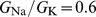 (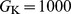 pS/*µ*m^2^ and 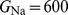 pS/*µ*m^2^) with a noise level of 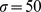 pA. The black lines denote the linear input-output relationships (slope 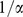) derived from the mean field which predict how the mean output firing rate of a given network layer can be derived from the mean input current into that layer, with the standard deviation of the prediction shown in gray. Note that within each of the four columns the slope of the linear prediction is identical, despite individual changes in the connectivity probability 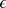 and the synaptic strength 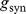. Three bottom row panels show the transmission of mean firing rates for a range of mean input currents as a function of layer number–each row of panels illustrates the outcome for different connection probability, 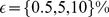, while also varying the strength of synaptic connectivity (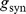) to preserve their product. Network parameters in each case: (weak, gain-scaling) 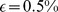, 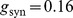; 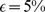, 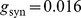, and 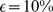, 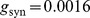, (strong, gain-scaling) 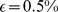, 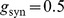; 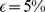, 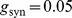, and 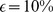, 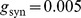, (weak, nongain-scaling) 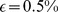, 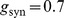; 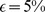, 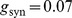, and 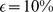, 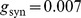, (strong, nongain-scaling) 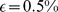, 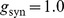; 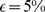, 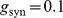, and 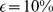, 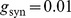. Although the firing rate profiles remain identical for 10% and 5% connectivity, as connectivity becomes sparser (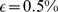) and stronger, the nongain-scaling network exhibits more asynchronous spiking and slow convergence to the fixed point of the mean field dynamics (bottom right panel). Compare to Figure 6 of the main text.(EPS)

Figure S3
**Firing rate transmission for a range of noise amplitudes** 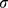 **.** Top: The 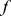
–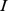 curves for gain-scaling and nongain-scaling networks with different 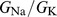 and for different levels of noise (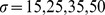, and 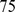 pA). We considered gain-scaling networks with 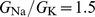 and **A**. weak vs **B**. strong-connectivity, **C**. gain-scaling networks with 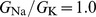 and strong connectivity, and **D**. nongain-scaling networks with 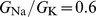 and strong connectivity (as weak connectivity was insufficient to drive activity in these networks). The black lines denote the linear input-output relationships derived from the mean field, which predict how the mean output firing rate of a given network layer can be derived from the mean input current into that layer with the standard deviation of the prediction shown in gray. The remaining panels show the transmission of mean firing rates across layers for a range of mean input currents–each row of panels illustrates the outcome for a different level of noise 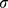. The results from numerical simulations over 10 second-long trials are shown as full lines (mean 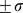 from 2000 neurons in each layer) and mean field predictions are shown in dashed lines with a shaded background in the same color (for each different input) illustrating the standard deviation of the prediction from the standard deviation in the linear input-output relationship in the top panels. Other network parameters: connection probability 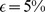, synaptic strength 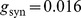 (weakly connected) and 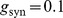 (strongly connected) and range of mean input firing rates 0–22 (for weakly connected) and 0–70 (for strongly connected). Compare to Figure 6 of the main text.(EPS)
